# Twist-tunable polaritonic nanoresonators in a van der Waals crystal

**DOI:** 10.1038/s41699-023-00387-z

**Published:** 2023-04-10

**Authors:** O. G. Matveeva, A. I. F. Tresguerres-Mata, R. V. Kirtaev, K. V. Voronin, J. Taboada-Gutiérrez, C. Lanza, J. Duan, J. Martín-Sánchez, V. S. Volkov, P. Alonso-González, A. Y. Nikitin

**Affiliations:** 1 Donostia International Physics Center (DIPC), 20018 Donostia/San Sebastián, Spain; 2 Department of Physics, University of Oviedo, 33006 Oviedo, Spain; 3 Center of Research on Nanomaterials and Nanotechnology, CINN (CSIC-Universidad de Oviedo), 33940 El Entrego, Spain; 4 XPANCEO, Bayan Business Center, DIP, 607-0406 Dubai, UAE; 5 IKERBASQUE, Basque Foundation for Science, 48013 Bilbao, Spain

**Keywords:** Optics and photonics, Two-dimensional materials, Two-dimensional materials

## Abstract

Optical nanoresonators are key building blocks in various nanotechnological applications (e.g., spectroscopy) due to their ability to effectively confine light at the nanoscale. Recently, nanoresonators based on phonon polaritons (PhPs)—light coupled to lattice vibrations—in polar crystals (e.g., SiC, or h-BN) have attracted much attention due to their strong field confinement, high quality factors, and their potential to enhance the photonic density of states at mid-infrared (mid-IR) frequencies, where numerous molecular vibrations reside. Here, we introduce a new class of mid-IR nanoresonators that not only exhibit the extraordinary properties previously reported, but also incorporate a new degree of freedom: twist tuning, i.e., the possibility of controlling their spectral response by simply rotating the constituent material. To achieve this result, we place a pristine slab of the van der Waals (vdW) α-MoO_3_ crystal, which supports in-plane hyperbolic PhPs, on an array of metallic ribbons. This sample design based on electromagnetic engineering, not only allows the definition of α-MoO_3_ nanoresonators with low losses (quality factors, Q, up to 200), but also enables a broad spectral tuning of the polaritonic resonances (up to 32 cm^−1^, i.e., up to ~6 times their full width at half maximum, FWHM ~5 cm^−1^) by a simple in-plane rotation of the same slab (from 0 to 45°). These results open the door to the development of tunable and low-loss IR nanotechnologies, fundamental requirements for their implementation in molecular sensing, emission or photodetection applications.

## Introduction

The excitation of PhPs in vdW crystals has recently emerged as an attractive strategy for manipulating IR light on deeply subwavelength scales^1–3^, enabling fingerprint identification of organic molecules as well as strong coupling phenomena^4,5^. Some of the PhPs, such as those excited in α-MoO_3_^6–15^ or α-V_2_O_5_^16^, present long lifetimes (up to a few ps) and in-plane hyperbolic propagation, being thus very appealing for the development of optical nanoresonators with ultra-low losses and potentially other unique properties. However, structuring of vdW crystals—needed for engineering nanoresonators—remains a challenging task, since the fabrication process dramatically degrades their optical properties, and thus the lifetime of PhPs^17^. Moreover, in contrast to plasmonic resonances in 2D materials, which are actively tunable via an external gate^18–20^, tunability of PhP nanoresonators is a more challenging task^21,22^. Here, we demonstrate PhP nanoresonators fabricated in the vdW crystal α-MoO_3_ that not only exhibit low losses, but also incorporate a unique twist tuning, i.e., the capability to be spectrally tuned by a simple rotation. The fabrication of these nanoresonators is based on placing a continuous (pristine) thin biaxial vdW crystal slab of α-MoO_3_ on top of a grating formed by metal nanoribbons (see Methods). Remarkably, such design avoids any degradation of the optical properties of the crystal due to fabrication, maintaining the intrinsic low-loss and in-plane hyperbolic propagation of PhPs in α-MoO_3_. The refractive indexes of the PhP modes supported by the α-MoO_3_ slab above the air and metal regions are different, so that propagating PhP modes can bounce back and forth between the boundaries of these regions (i.e., α-MoO_3_/air and α-MoO_3_/metal), eventually forming resonances. Most importantly, since the in-plane hyperbolicity of α-MoO_3_, implies that different in-plane directions support PhPs with different momenta, the mutual orientation of the crystal axes and the metal grating can serve as a tuning knob of such resonances. Thus, by twisting always the same α-MoO_3_ slab with an in-plane angle with respect to the metal ribbons, we demonstrate a broad tunability of the PhP nanoresonators, as revealed by both far- and near-field techniques: Fourier-transform IR spectroscopy (FTIR) and scattering-type scanning near-field optical microscopy (s-SNOM), respectively. Furthermore, with the help of theoretical analysis, we are able to interpret and disentangle the different resonant PhP modes observed in the experimental data.

## Results and discussion

**Fig. 1:**
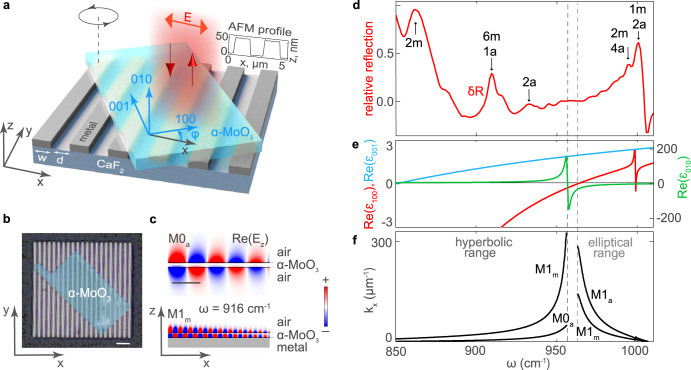
PhP nanoresonators in α-MoO_3_ defined by placing a pristine α-MoO_3_ slab on metallic ribbons. **a** Schematics of the studied structure that allows defining the nanoresonators by “electromagnetic engineering” and controlling them by a twist angle, $\varphi$. The inset shows the AFM profile of the metal grating. **b** False-color optical image (top view) of the sample consisting of a 110 nm-thick α-MoO_3_ slab placed on a 50 nm-thick metal grating. Scale bar: 8 μm. **c** Simulated field distributions of the M0 and M1 PhP modes in the α-MoO_3_/air (top) and α-MoO_3_/metal (bottom) regions. **d** Relative far-field reflection spectrum of the PhP nanoresonators for $\varphi = 0$°. The subscripts “a” and “m” indicate the PhP resonances originated in the α-MoO_3_/air and α-MoO_3_/metal regions, respectively. **e** Real part of the dielectric permittivity tensor components as a function of $\omega$. **f** Calculated dispersion of the M1 and M0 modes shown in (**c**).

Figure 1a illustrates schematically the heterostructure designed to define “electromagnetic-engineered” PhP nanoresonators in our work. It consists of a single α-MoO_3_ slab placed on top of a periodic array of metal ribbons fabricated on an IR-transparent CaF_2_ substrate (see the AFM profile in the inset to Fig. 1a and the optical image in Fig. 1b), similar to the concept of polaritonic nanoresonators based on in-plane isotropic slabs of h-BN and graphene layers^23–25^. The α-MoO_3_ slab is twisted with respect to the direction across the ribbons (x-direction) by an arbitrary angle $\varphi$. The metal arrays (with ribbon widths $w = 1480$ nm and a separation distance $d = 1230$ nm) have been designed so that the heterostructure exhibits PhP resonances at mid-IR frequencies (in the range $\omega = 850 - 1010$ cm^−1^). The reflection spectrum taken by FTIR upon incident illumination polarized across the ribbons and along the α-MoO_3_ [100] crystal direction ($\varphi = 0$°) is illustrated in Fig. 1d. To better recognize the resonance spectral features, we normalize the reflection coefficient, *R*, to its moving average, $\bar R$, obtaining the relative reflection $\delta R = (R - \bar R)/\bar R$ (Fig. 1d, red curve), which clearly demonstrates a set of sharp resonance peaks (even though the spectral normalization was performed by comparing to a bare Au substrate). Particularly, these peaks appear within the Reststrahlen Bands (RBs) of the α-MoO_3_ crystal (Fig. 1e), defined in the spectral ranges $\omega = 850 - 956.8$ cm^−1^, where ${{{\mathrm{Re}}}}\left( {\varepsilon _{100}} \right) \,< \,0$, and $\omega = 963 - 1006$ cm^–1^, where ${{{\mathrm{Re}}}}\left( {\varepsilon _{010}} \right) \,<\, 0$, exhibiting Q as large as 180 at 916 cm^−1^ and 200 at 993 cm^−1^ (see Supplementary Note V). In the lower (upper) RB PhPs exhibit hyperbolic (elliptic) dispersion^26^, composing a set of in-plane anisotropic electromagnetic modes propagating along the crystal slab. Importantly, the PhP modes in the region α-MoO_3_/air (M*l*_a_) are expected to have a different refractive index (momentum) to those in the region α-MoO_3_/metal (M*l*_m_), even for the same mode number, *l*, the latter representing the quantization of the PhP’s electric field in the z-direction^9,27^. This is corroborated by plotting the dispersions of the fundamental modes in both regions within the hyperbolic and elliptic RBs (M0_a_, M1_a_ and M1_m_ in Fig. 1f, with modes corresponding to the α-MoO_3_ [100] crystal direction). Clearly, the M1_m_ mode exhibits a much shorter wavelength (larger momentum), together with stronger field confinement to the faces of the α-MoO_3_ slab (Fig. 1c). We thus assume that the resonant features seen in the spectra of Fig. 1d correspond to Fabry-Pérot resonances (FPR) arising from multiple reflections of the PhP waveguide modes due to the mode’s refractive index steps defined along the x-axis.

### Simulated and measured reflectance spectra of the nanoresonators

**Fig. 2:**
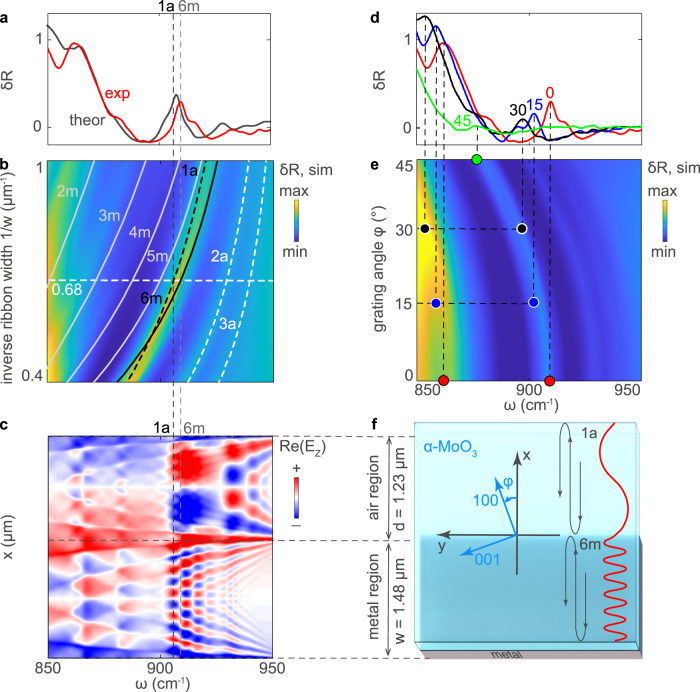
Analysis of the PhP resonances and their twist tuning. **a** Measured and simulated relative reflection spectra, $\delta R$ (solid red and gray curves, respectively), for a ribbon width $w = 1480$ nm, a separation distance $d = 1230$ nm and a twist angle $\varphi = 0^\circ$. The flake thickness is 110 nm. **b** Simulated $\delta R$ as a function of frequency, $\omega$, and inverse ribbon width, $1/w$, for a fixed ratio $w/d$. The FPR conditions for PhP in α-MoO_3_ above the air and metal regions are traced by the white solid and dashed lines, respectively. **c** Simulated field distributions of the M0 and M1 PhP modes in the α-MoO_3_/air and α-MoO_3_/metal regions, respectively, as a function of $\omega$ and the $x$ coordinate (short axis of the ribbons). **d** Measured $\delta R$ spectra for twist angles of the same α-MoO_3_ slab $\varphi = 0,\,15,\,30$ and 45° (red, blue, black and green, respectively). **e** Simulated relative reflection as a function of $\omega$ and $\varphi$. **f** Top view of one lattice unit cell.

To corroborate our assumption on the physical origin of the PhP resonances and explain the reflection spectra obtained experimentally, we perform a theoretical analysis based on full-wave numerical simulations of a structure mimicking the fabricated nanoresonators. The simulated relative reflection spectrum (gray curve in Fig. 2a) shows a good agreement with the experiment (red curve in Fig. 2a) for the case of $w = 1480$ nm and $d = 1230$ nm. We, therefore, extend the theoretical study for a broad range of *w* and *d* in Fig. 2b, where $\delta R$, represented by a color plot, shows a set of maxima that clearly depend on the ribbon width or the distance between the ribbons. These resonances presumably coincide with an anisotropic FPRs condition, which can be written in a simple form according to the PhP mode phase matching as $a \cdot k_M + \phi _M = 2\pi n$, analogously to that for in-plane isotropic FPRs in graphene ribbons^25,28^. In our case, *a* coincides with either *w* or *d*, depending on the region in the α-MoO_3_ slab where the nanoresonators are defined (above the metal or air, respectively), $k_M$ and $\phi _M$ are the momentum and reflection phase of the *M*th propagating waveguide mode of the slab, respectively, and *n* is the integer numerating the FPRs (quantization of the *M*th PhP mode in the x-direction), indicating how many wavelengths of the mode fit across either the distance *w* or *d*. This numeration includes only the “bright” FPRs, corresponding to the non-zero overlap between the illuminating wave and the PhPs in a FPR cavity^28^. Plugging the $k_M$ from the dispersion relation of PhP modes in a biaxial crystal slab^9,27^ into the FPR condition, we obtain a set of curves for each of the modes (rendered on top of the color plot in Fig. 1b). These curves match the maxima of $\delta R$, thus confirming the Fabry-Pérot origin of the resonances in our polaritonic structures. The FPR can also be recognized in the calculated spatial distribution of the vertical electric field above the unit cell of the periodic structure (see schematics in Fig. 2f), represented as a function of frequency $\omega$ and coordinate *x* in Fig. 2c. Indeed, at the peak frequencies the number of field oscillations across the ribbon matches with the quantization number *n* in the FPR condition, both for the PhP modes in the α-MoO_3_/air regions (marked with “*n*a”) and in the *α*-MoO_3_/metal regions (marked with “*n*m”).

Importantly, the spectral position of the PhP resonances strongly depends on the twist angle $\varphi$ between the *α*-MoO_3_ slab and the metallic ribbons, as clearly observed in the reflection spectra of Fig. 2d. In particular, the main resonant peak (1a) shifts to lower frequencies (from ~909.5 to ~877 cm^−1^) with increasing $\varphi$ (the increasing signal-to-noise ratio enables determining the resonant features for $\varphi$ up to 45°), which can also be seen in our theoretical calculations (Fig. 2e). Note the much higher Q (see Supplementary Note V) of our twist-tunable PhP resonators (by more than an order of magnitude, due to both the absence of the electron scattering and avoiding the direct crystal structuring) compared to their plasmonic counterpart reported at THz frequencies in in-plane anisotropic WTe_2_ crystals^29^.

### Nanoimaging of the nanoresonators by s-SNOM

**Fig. 3:**
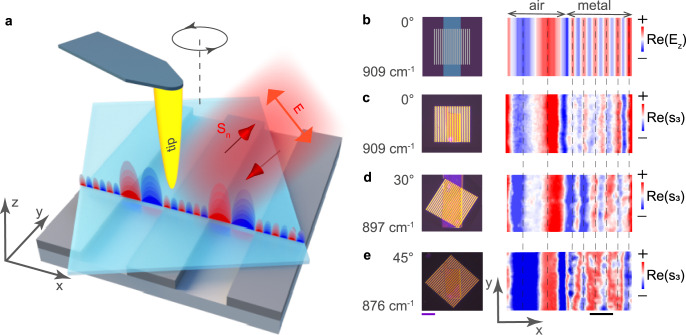
Near-field measurements of twist-tunable PhP nanoresonators. **a** Schematic of the near-field measurements by s-SNOM, in which a metal tip is illuminated by p-polarized mid-IR light and both the amplitude and phase of the tip-scattered field are recorded as a function of the tip position. **b** Simulated field distribution, Re($E_z$), in the α-MoO_3_/air and α-MoO_3_/metal regions. **c**–**e** Experimental near-field images, Re(*s*_3_), of one of the unit cells of the heterostructure for the twist angles $\varphi = 0,\,30,$ and 45°, respectively, taken at the resonant frequencies. The flake thickness is 110 nm since we use the same flake in all the experiments. Purple scale bar: 20 μm and black scale bar: 0.5 μm.

To further corroborate our results and the structure of the electric field in the anisotropic PhP nanoresonators, we perform near-field measurements by s-SNOM (see Methods). By scanning the α-MoO_3_ slab with the s-SNOM tip (Fig. 3a), we record the electromagnetic signal as a function of the tip position, composing the near-field images (Fig. 3c–e). At a frequency $\omega = 909$ cm^−1^ (Fig. 3c), corresponding to the 1a and 6 m resonances in the far-field spectra (Fig. 2a), we observe signal fringes across the ribbons, indicating the excitation of PhPs. The much larger distance between the red and blue fringes in the region α-MoO_3_/air (1 oscillation) compared to the region α-MoO_3_/metal (6 oscillations) is consistent with the much longer PhP wavelength (see the simulated PhP field distribution along the black and gray vertical dashed lines, “1a, 6m”, in Fig. 2c), as expected from their dispersion relation (Fig. 1f). For better visualization, we represent the simulated Re($E_z$) as a function of the in-plane coordinates $x,y$ in Fig. 3b, which matches well with the near-field image in Fig. 3c. Note that although the fringe spacing is fairly constant, the number of fringes is not exactly the same in all cases, which we attribute to a non-negligible contribution from tip-launched PhPs. From these observations, we can thus conclude that: (i) the s-SNOM tip-scattered signal represents a measure of Re($E_z$) above the slab^17,30^ and (ii) the visualized near-fields at the resonant peaks of the far-field spectra are clearly consistent with the FPR model. Remarkably, changing both the measuring frequency and the twist angle of the same α-MoO_3_ layer, following the experimental data shown in Fig. 2d, the number of oscillations in the near-field images keeps constant (the agreement between the periods of the near-field signal oscillations across the ribbons is shown in Fig. 3c–e by the vertical dashed eye-guides). Namely, the calculated PhPs wavelength in the area above the air gap (1a resonance) is equal to $\lambda _p = 1.29$ μm, approximately fitting the air gap width (1.23 μm), while in the area above the metal (6m resonance) $\lambda _p = 249$ nm, thus being consistent with the ribbon width divided by 6 (247 nm). Therefore, our near-field measurements reconfirm the emergence of FPR of the same order for the chosen parameters. On the other hand, although similar oscillations are observed in the near-field images, the FPR originates from PhPs at different points in the dispersion surface (oscillating at different frequencies and propagating along different directions).

### Reconstruction of the isofrequency curves from the Fabry-Pérot resonances

**Fig. 4:**
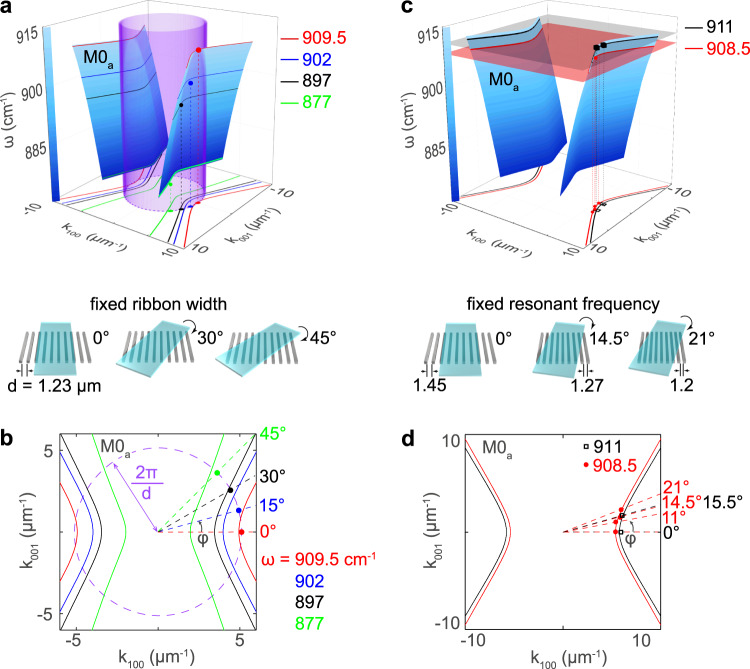
Probing the dispersion surface of the M0_a_ PhP mode. **a** Analytical dispersion surface of the M0_a_ PhP mode (blue hyperboloid) when crossed by a surface (purple cylinder) representing a constant momentum. The color dots represent the positions of the measured resonant peaks (from Fig. 2d) for the same α-MoO_3_ slab and metal grating twisted at different angles, $\varphi$. **b**, **d** Isofrequency curves for the M0_a_ mode at different frequencies $\omega$. **c** Analogous dispersion surface as in (**a**), but crossed by planes of fixed frequencies ($\omega = 908.5$, and $911$ cm^−1^). The points mark the peak positions for metal lattices with different widths of the air gaps *d* and twist angles $\varphi$. The thicknesses of the flakes in (**a**, **b**) and (**c**, **d**) are 110 and 127.5 nm, respectively.

As mentioned above, we assume that the resonances are built upon multiple reflections of propagating waveguide PhP modes in the slab. In order to illustrate the “probing” of one of these modes (M0_a_) in our nanoresonators at different points of the dispersion surface, we mark the positions of the main (1a) peaks in the normalized reflection spectra (Fig. 2d) directly on the hyperboloids representing the dispersion of the M0_a_ PhP mode (Fig. 4a). The experimentally-measured positions of the resonances (green, black, blue and red points in Fig. 4a) can be represented in polar coordinates by the frequency, $\omega$, of the resonant peak (z coordinate), the inverse air gap width, $2\pi /d$ (radial coordinate), and the twist angle, $\varphi$. The selection of the PhPs momenta for the first-order FPR at any $\varphi$ can be visualized by a cylinder with a radius of $2\pi /d$, passing through the dispersion surface (Fig. 4a). Crossings between the latter and the cylinder give the approximate positions of the FPR, which can be more clearly seen in the $k_x,k_y$ plane (Fig. 4b), where the isofrequency curves (IFCs) for each resonant frequency are shown. The experimental points fall very close to the crossings between the circle and the IFCs, thus reconfirming our initial assumption on the Fabry-Pérot origin of the resonances.

Alternatively, the PhP dispersion surface can also be probed by cutting it with planes of constant frequencies, $\omega$. To that end, we performed an additional set of far-field measurements for metal gratings with different air gaps, *d*, and twist angles, $\varphi$, all of them matching the same FPR (see schematics in Fig. 4). Namely, we designed our structures to exhibit the “1a” FPR at two fixed frequencies: 908.5 cm^−1^ and 911 cm^−1^ (see measurements in the Supplementary Note I). The positions of the measured resonant peaks perfectly match with the crossing points between the calculated IFCs and the radial lines from the origin at an angle $\varphi$ (dashed lines in Fig. 4d), as seen in Fig. 4c, d for both frequencies. Note that a similar analysis can be also easily performed for the FPRs of other orders, and particularly for the FPRs composed by the PhP modes above the metallic region, M1_m_ (see Supplementary Note III). This analysis is equally suitable for the upper RB, i.e., for the elliptic regime (see Supplementary Notes II and IV).

To summarize, we introduce Fabry-Pérot nanoresonators that exhibit a unique tuning by a simple rotation of the host crystal. This is achieved by fabricating a heterostructure composed of a metal grating and a single vdW crystal slab supporting in-plane anisotropic PhPs (α-MoO_3_) that is rotated in the plane. In contrast to conventional Fabry-Pérot polaritonic resonators, in which the reflecting boundaries are typically fabricated by etching the polaritonic material, the design of our tunable Fabry-Pérot nanoresonators allows preserving the crystalline properties of the slab and thus obtaining high Q factors. Interestingly, the resulting FPRs, visualized in real space by s-SNOM, allow reconstruction of the three-dimensional anisotropic dispersion surfaces of the PhPs from data collected at either fixed momentum or fixed frequency. Such reconstruction of the polaritons dispersion opens the door to an alternative characterization of new vdW materials, particularly for a more reliable determination of their optical properties. From an application point of view, our nanoresonators can be very appealing for tunable mid-IR narrow-resonance sensors, and in particular for molecular bar-coding^31^.

## Methods

### Fabrication of metal ribbons

Arrays of ribbons of both Au and Al were fabricated by electron beam lithography. In the case of Al ribbons, first, a 70 nm-thick Al layer was deposited by electron beam evaporation on a CaF_2_ substrate. Subsequently, a negative photoresist layer (MA-N2401) was spin-coated (3000 rpm -> 90 nm) on top of the Al layer for the definition of the ribbon pattern by electron beam lithography (50 keV, 200pA, dose 280 uC/cm^2^). A high-resolution developer AZ726 was used for the lift-off. By reactive-ion etching of Al in BCl_3_/Cl_2_ plasma (pressure 40mT, RIE power 100 W) and removing the photoresist in oxygen plasma, we got the set of 50 μm (length) × 60 μm (width) × 70 nm (height) gratings with different ribbon widths. In the case of Au ribbons, high-resolution electron beam lithography was employed under 100 kV and 100 pA. First, the samples were coated with a positive resist layer (PMMA). To dissolve the exposed areas, a conventional high-resolution developer (1:3 MIBK: IPA) was used. Afterward, 5 nm of Cr and 50 nm of Au were evaporated. Finally, the lift-off was performed to define the 50 µm (length) × 50 µm (width) × 50 nm (height) Au grating with ribbons of 1.48 µm width.

We used the set of Al gratings exclusively for measurements presented in Fig. 4c, d. For all other measurements, we used the Au grating.

### Fabrication and in-plane twist of α-MoO_3_ flakes

α-MoO_3_ flakes were mechanically exfoliated using a Nitto tape (Nitto Denko Co., SPV 224 P) from commercial α-MoO_3_ bulk crystals (Alfa Aesar). A second exfoliation was performed from the tape to transparent polydimethylsiloxane (PDMS) in order to thin them down. The flakes were examined with an optical microscope in order to select homogeneous pieces with the desired thicknesses (110 nm and 127.5 nm) and large surface areas. For twisting them the dry transfer technique was used, with the help of a micromanipulator, the flakes were precisely aligned and twisted on top of the metal grating, picked up with the help of polycarbonate (PC). Transferring was carried out by heating up to 250 °C in order to liquate the PC. The PC was removed with chloroform at 100 °C releasing the flake.

### Fourier-transform infrared spectroscopy

The far-field optical response of our α-MoO_3_ nanoresonators was characterized by Fourier-Transform Infrared Spectroscopy (FTIR) using a Varian 620-IR microscope coupled to a Varian 670-IR spectrometer, supplied with a broadband mercury cadmium telluride (MCT) detector (400–6000 cm^−1^). The reflectance spectra were collected with a 2 cm^−1^ spectral resolution and the spatial resolution was adjusted to the size of the metal arrays. The infrared radiation from the thermal source (normal incidence) was linearly polarized employing a wire grid polarizer. CaF_2_ substrates were used due to their transparency in the spectral range under study. An Au layer is used as a reference for normalization.

### Scattering-scanning near-field optical microscopy

Near-field imaging measurements were performed by employing a commercial scattering-type Scanning Near-field Optical Microscope (s-SNOM) from Neaspec GmbH, equipped with a quantum cascade laser from Daylight Solutions (890–1140 cm^−1^). Metal-coated (Pt/Ir) atomic force microscopy (AFM) tips (ARROW-NCPt-50, Nanoworld) at a tapping frequency $\Omega$ ~280 kHz and an oscillation amplitude ~100 nm were used as source and probe of polaritonic excitations. The light scattered by the tip was focused by a parabolic mirror into an infrared detector (Kolmar Technologies). Demodulation of the detected signals to the 3rd harmonic of the tip frequency (*s*_*3*_) was carried out for background suppression. A pseudo-heterodyne interferometric method was employed to independently extract both amplitude and phase signals.

### Full-wave numerical simulations

Two types of full-wave numerical simulations were performed using COMSOL software, based on the finite-element method in the frequency domain. In both cases, the structure was composed of the α-MoO_3_ flake on top of the array of metal ribbons placed on the semi-infinite CaF_2_ substrate. The first type of simulation was based on the far-field illumination of the structure by a normally-incident plane wave polarized across the ribbons. The ratio of the ribbon to the air gap widths was fixed constant $w/d = 1480/1230$ (the experimental measurements for the array of ribbons with such parameters were performed in Figs. 1d, 2a and 3b–f). The reflection coefficient and the spatial distribution of the vertical electric field, $E_z$, above the α-MoO_3_ slab have been extracted from the simulations (see Fig. 2a–e, Fig. 3b–e). In the second type of simulation, we have used the quasi-normal eigenmode analysis in order to find the electric field distribution of the modes M0 and M1 (see Fig. 1c).

In the theoretical analysis of the FPR (Fig. 2b and Fig. 4), the reflection phase of the PhP modes, $\phi _M$, in the α-MoO_3_/metal and α-MoO_3_/air regions is taken 0 and π, respectively.

### Supplementary information

Supplementary Information for “Twist-tunable polaritonic nanoresonators in a van der Waals crystal”

## Data Availability

The authors declare that the data supporting the findings of this study are available within the paper and its supplementary information. The corresponding author can also provide data upon reasonable request.
