# Supplementary material for: Twist-tunable polaritonic nanoresonators in a van der Waals crystal
Source: NPJ 2D Mater Appl. 2023 Apr 10;7(1):31. doi: 10.1038/s41699-023-00387-z (PMC11041695; doi:10.1038/s41699-023-00387-z)
Supplement: Supplementary file 1 — Supplementary Information for “Twist-tunable polaritonic nanoresonators in a van der Waals crystal” [file 41699_2023_387_MOESM1_ESM.docx]

**Supplementary Information for “Twist-tunable polaritonic nanoresonators in a van der Waals crystal”**

O. G. Matveeva^1†^, A. I. F. Tresguerres-Mata^2†^, R. V. Kirtaev^1^, K. V. Voronin^1^, J. Taboada-Gutiérrez^2,3^, C. Lanza^2^, J. Duan^2,3^, J. Martín-Sánchez^2,3^, V. S. Volkov^4^, P. Alonso-González^2,3^*, A. Y. Nikitin^1,5^*

*^1^Donostia International Physics Center (DIPC), Donostia/San Sebastián 20018, Spain*

*^2^Department of Physics, University of Oviedo, Oviedo 33006, Spain.*

*^3^Center of Research on Nanomaterials and Nanotechnology, CINN (CSIC-Universidad de Oviedo), El Entrego 33940, Spain.*

*^4^XPANCEO, Bayan Business Center, DIP, 607-0406, Dubai, UAE*

*^5^IKERBASQUE, Basque Foundation for Science, Bilbao 48013, Spain.*

[*alexey@dipc.org](mailto:*alexey@dipc.org) [pabloalonso@uniovi.es](mailto:pabloalonso@uniovi.es)

^†^*These authors contributed equally to this work.*

**Table of Contents**

Supplementary Note I. Far-field Characterization……………………….………………3

Supplementary Note II. Analysis of the Anisotropic PhP Resonances in the Elliptic α-MoO_3_ Frequency Range………………………………...……………………………..…4

Supplementary Note III. Dispersion Surface of the M1_m_ PhP Mode above the Metal Ribbon Regions in the α-MoO_3_ Hyperbolic Frequency Range…………………………..7

Supplementary Note IV. Dispersion Surfaces of the M1_a_ and M1_m_ PhP Modes above both the Air Gap and the Metal Ribbon Regions in the α-MoO_3_ Elliptic Frequency Range…...9

Supplementary Note V. Experimental Quality Factors of the Nanoresonators…….........10

Supplementary References……………..……………......……………………………...12

In this Supporting Information, we will consider the Fabry- Pérot resonances (FPRs) with their origin in: i) the M1 PhP mode propagating in the α-MoO_3_ flake above the metal region in the hyperbolic frequency range, and ii) the M1 PhP mode propagating in the flake above both the metal ribbon and the air gap regions in the elliptic frequency range.

**Supplementary Note I. Far-field Characterization**

The far-field characterization of our samples was carried out by performing FTIR measurements on an α-MoO_3_ flake with thickness *t* = 110 nm placed on top of Au ribbons with a width *w* = 1.48 μm and a separation *d* = 1.23 μm (Supplementary Figure 1a). Within the hyperbolic frequency range we have identified the FPRs 1a and 6m, matching the same resonance frequency ( = 909.5 cm^-1^, see Figure 1d of the main text). In contrast, in the elliptic range, we have identified the resonances 2m and 4a close to  = 995 cm^-1^ (Figure 1d in the main text). The analysis of the resonances 2m and 4a for different twisting angles $\varphi$ is presented in Supplementary Figure 3d of Supplementary Note II.

| 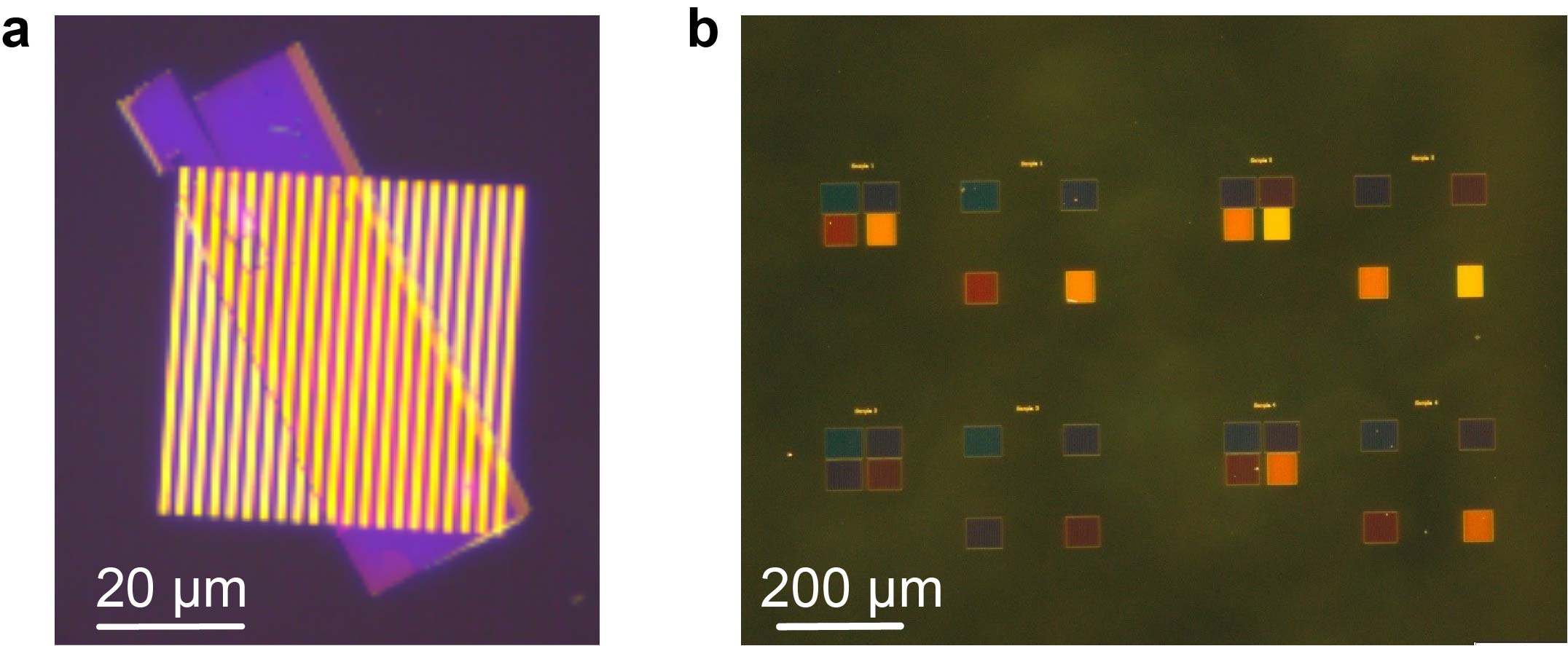 |
| --- |
| **Supplementary Figure 1. Optical images of the metal gratings. a)** Au grating with a ribbon width *w* = 1.48 μm and a separation (air gaps) *d* = 1.23 μm. The __ flake placed on top of them has a thickness *t* = 110 nm. **b)** Sets of Al gratings with different ribbon widths and air gaps. The substrate is in all cases CaF_2_. |

Additionally, we fabricated Al gratings (as the type of metal does not affect our results in the mid-IR, in some cases we use Al instead of Au for convenience) with different periods (Supplementary Figure 1b). In this case, the *α*__ flake placed on top had a thickness of *t* = 127.5 nm. By varying the twisting angle of the flake with respect to the ribbons axes, we observe the same resonant frequencies as when using Au in both hyperbolic and elliptic frequency ranges (Supplementary Figure 2a,b, and Supplementary Figure 2c, respectively). The FPRs modes 1a and 6m in the hyperbolic range appear at  = 908.5 cm^-1^ (Supplementary Figure 2a) and  = 911 cm^-1^ (Supplementary Figure 2b), while the FPRs 2m and 4a in the elliptic range appear at  = 991 cm^-1^ (Supplementary Figure 2c). The ribbons and air gaps widths were measured by AFM.

| 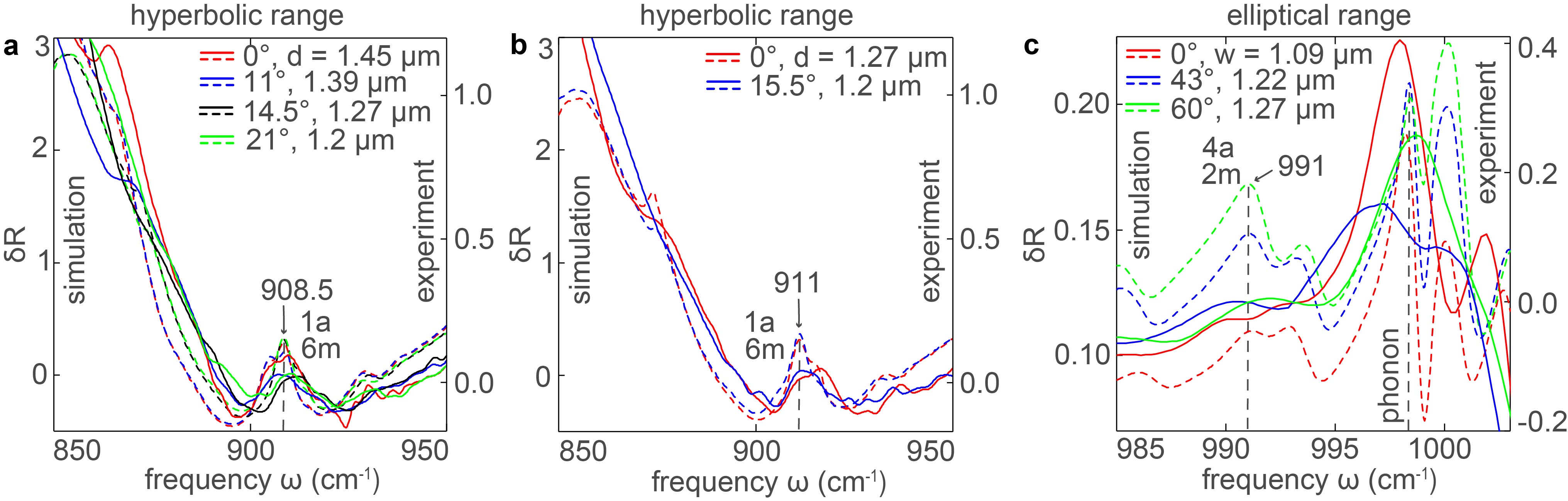 |
| --- |
| **Supplementary Figure 2.** **Far-field spectra of PhP nanoresonators in the case of using Al gratings. a)** Experimental and simulated relative reflection spectra in the hyperbolic range for different air gap widths, *d,* and flake rotation angles, $\varphi$, showing resonances (FPRs 1a and 6m for the M0 and M1_m_ PhP modes, respectively) at the same frequency, ω = 908.5 cm^-1^. **b)** Analogous spectra as in (a) for gratings with a resonant frequency ω = 911 cm^-1^. **c)** Experimental and simulated relative reflection spectra in the elliptic range for nanoresonators defined on gratings with different ribbon widths *w* and twisting angles $\varphi$. The nanoresonators show a maximum of their relative reflection at the same frequency ω = 991 cm^-1^ (resonances 2m and 4a for the M1_m_ and M1_a_ PhP modes, respectively). Solid and dash lines in (a-c) represent experimental and simulated data, respectively. The thickness of the _ _flake was 127.5 nm. |

Supplementary Figures 2a-c show the relative reflection spectra for an α-MoO_3_ flake placed on top of Al gratings with different air gaps (Supplementary Figures 2a,b) and ribbon widths *w* (Supplementary Figure 2c). For all the gratings the flake was rotated by a given angle $\varphi$ such that the resonances 1a and 6m (Supplementary Figures 2a,b), as well as the resonances 2m and 4a (Supplementary Figure 2c) take place at the same frequency.

**Supplementary Note II. Analysis of the Anisotropic PhP Resonances in the Elliptic α-MoO_3_ Frequency Range**

The FTIR relative reflection spectrum (red dots in Supplementary Figure 3a), hereinafter referred to as *δR*, of nanoresonators in the elliptic α-MoO_3_ frequency range was measured for a 110nm-thick α-MoO_3_ flake placed on top of an array of Au-ribbons (with a period of 2.71 μm and a ribbon width of 1.48 μm, corresponding to *d* = 1.23 μm) at *φ* = 0°. For comparison, full-wave numerical simulations of *δR* (black curve in Supplementary Figure 3a) were carried out mimicking the experiment (in both cases using plane wave illumination polarized across the ribbons). Although the agreement between the experiment and theory is reasonably good, the spectral resolution in our FTIR measurements (2 cm^-1^) is too low to capture the TO-phonon along the [100] crystal direction at  = 998.7cm^-1^. The color plot in Supplementary Figure 3b shows *δR* as a function of frequency and inversed ribbon width (while the ratio between the ribbon width and the width of the air gap is fixed at 1.48/1.23).

In our system, we consider the FPRs in both regions of the flake (above the metal and air) as independent (i.e. completely neglecting any coupling between them). In this approximation, we can write the phase-matching conditions^1^:

*k*_a_*g + Ф*_a_*= 2πn*a(1)

*k*_m_*w + Ф*_m_*= 2πn*m,(2)

where *k_a_*and *k*_m_ are the wavevectors in the regions above the air gaps and the metal ribbons, *Ф*_a_and *Ф*_m_are the reflection phases from the air/metal and metal/air boundaries, and *n*aand*n*mrepresent the number of polariton wavelengths fitting in the areas above the air gaps and metal ribbons, respectively. We take the wavevectors in Eqs. (1,2) from the analytical equation for the dispersion of anisotropic modes M*l* in a thin biaxial slab of thickness *d*, propagating in the plane at an angle *φ* with respect to the [100] crystallographic direction^2^:

$k\left( \omega\right)= \frac{\rho}{k_{0}d}$[arctan($\frac{\varepsilon_{1}\rho}{\varepsilon_{z}}$) + arctan($\frac{\varepsilon_{1}\rho}{\varepsilon_{z}}$) + π*l*], *l* ∈ ℤ (3)

$\rho=i\sqrt{\frac{\varepsilon_{z}}{\varepsilon_{x}{cos}^{2}\varphi+ \varepsilon_{y}{sin}^{2}\varphi}}$.

For simplicity, we neglect the reflection phases (*Ф*_a_ = *Ф*_m_ = 0). For each number *n*a and *n*m and fixed relation *w/d*, the wavevector *k*_a,m_ is a function of a single parameter, *w*, which we represent by the solid and dashed curves in Supplementary Figure 3b (labeled as “*n*a” and “*n*m”, respectively).

Analogously to the case analyzed in the main text for the hyperbolic α-MoO_3_ spectral range, for the elliptic spectral range we identified the specific FPRs corresponding to the peak positions in the *δR* spectrum in Supplementary Figure 3a. To that end, we monitor the intersection between a horizontal line representing the inverse ribbon width (1.48μm) and the dispersion curves (Supplementary Figure 3b). In the experimental spectrum, the resonant peaks at the frequencies matching *n*a = 2*n*m show the highest intensity. This can be explained by the 2 times larger PhP wavevectors of the M1 mode in the areas above the air gaps compared to those above the metal ribbons (due to the effective doubling of the flake thickness caused by the mirror effect of the metal). In contrast, in the hyperbolic range, the modes are intrinsically different: while the regions above the air gaps support the M0 mode, the regions above the metal ribbons support the M1 mode. Consequently, the doubling effect is not observed in the hyperbolic frequency range. Note that the M0 mode in the flake above the air gaps in the elliptic range is not supported since the dielectric permittivities *ε_x_* and *ε_y_* are both positive.

Supplementary Figure 3c shows the real part of the out-of-plane component of the vertical electric field across the ribbons as a function of frequency. The number of field oscillations across the ribbon coincides with the resonance numbers, *n*a and *n*m, labeling the resonance peak in Supplementary Figure 3a. In Supplementary Figure 3d, we illustrate the measured *δR* spectra for $\varphi=$0°, 15°, 30°, and 45°, in which the positions of the resonances 2m and 4a agree well with the simulated spectra for all angles $\varphi$ (color plot in Supplementary Figure 3e). Thus, we also see a strong dependence of the resonant frequencies on the rotation angles within the elliptic range, both in the experiment and theory.

| 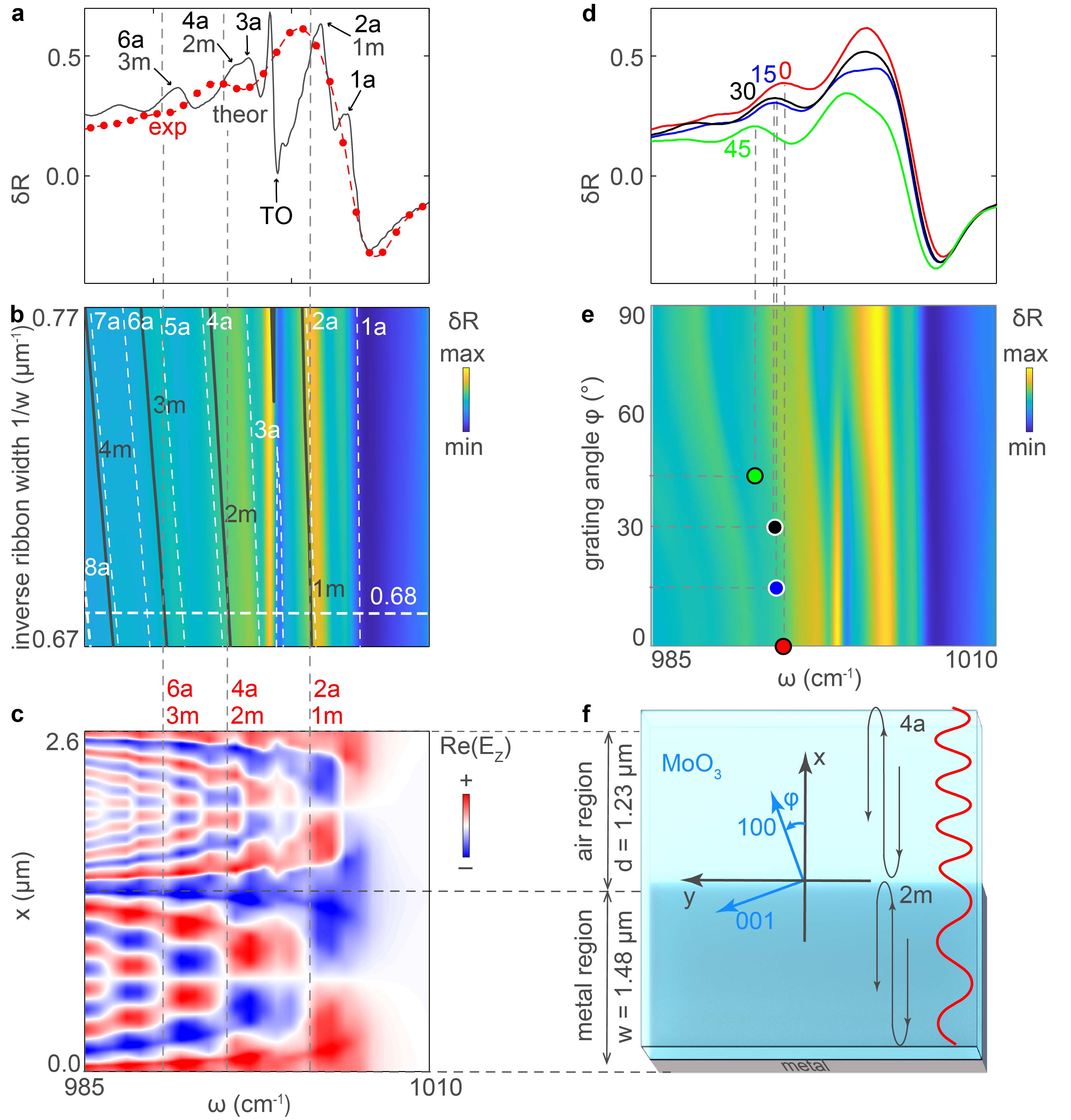**Supplementary Figure 3. Analysis of the PhP FPRs in the elliptical range and their twist-tuning. a)** Measured and simulated relative reflection spectra, $\delta R$ (dash red and solid grey curves, respectively), for a ribbon width *w* = 1480 nm, separation distance *d* = 1230 nm, and twist angle $\varphi=0^{\circ}$. **b)** Simulated $\delta R$ as a function of frequency *ω* and the inverse ribbon width, 1/*w*, for a fixed ratio *w*/*d*. **c)** Simulated field distributions of the M1_a_ and M1_m_ PhP modes in the α-MoO_3_/air and α-MoO_3_/Au regions, respectively, as a function of *ω* and the *x* coordinate (across the ribbons). **d)** Measured $\delta R$ spectra for the twist angles $\varphi=$0, 15, 30, and 45° (red, blue, black, and green, respectively). **e)** Simulated relative reflection as a function of *ω* and $\varphi$. **f)** Schematics of the top view of one lattice unit cell. |
| --- |

**Supplementary Note III. Dispersion Surface of the M1_m_ PhP Mode above the Metal Ribbon Regions in the α-MoO_3_ Hyperbolic Frequency Range**

| 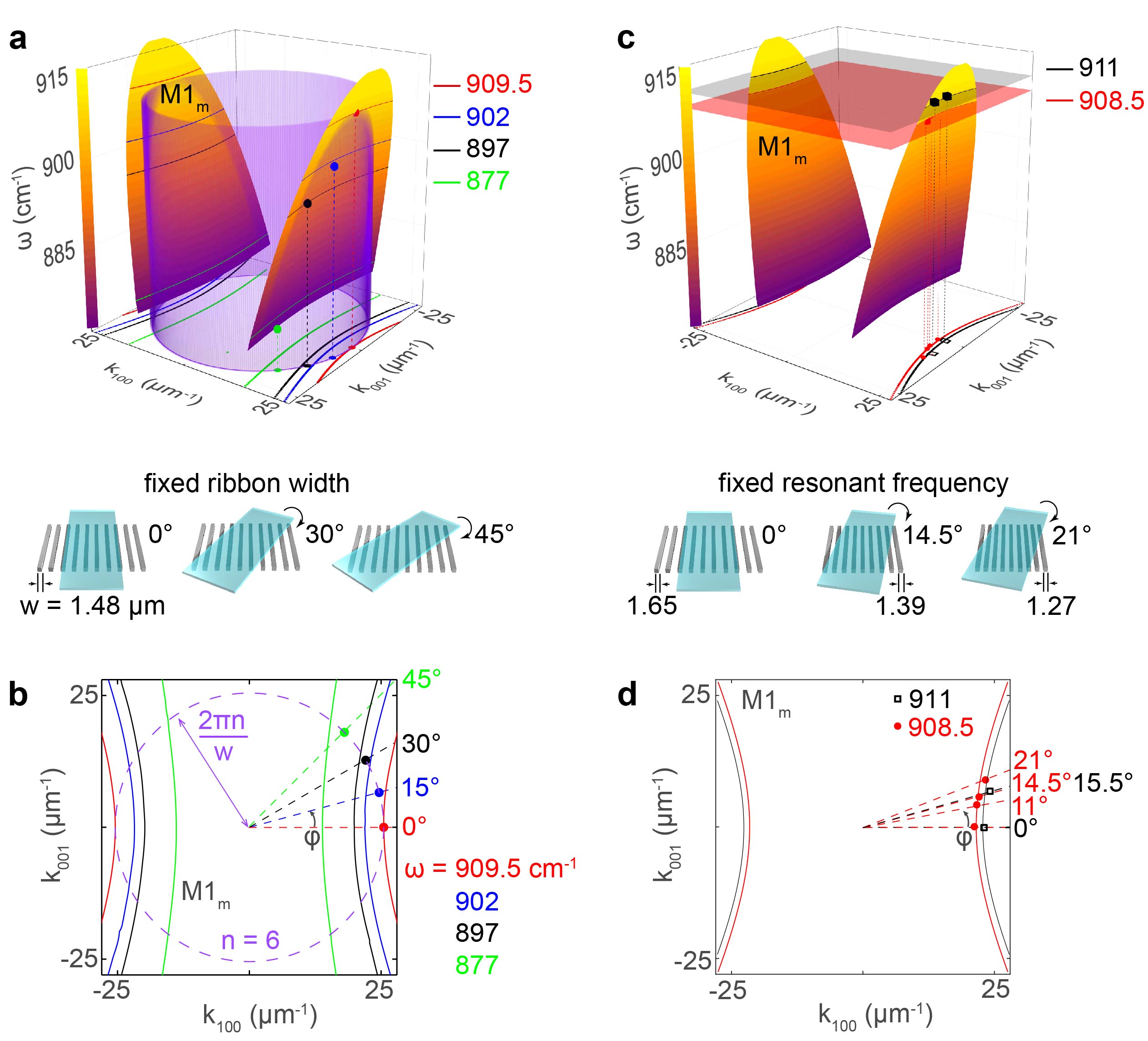 |
| --- |
| **Supplementary Figure 4.** **Probing the dispersion surface of the M1_m_ PhP mode in the hyperbolic regime. a)** Analytical dispersion surface of the M1_m_ PhP mode crossed by a cylinder representing a constant momentum. The color dots represent the positions of the measured resonant peaks 6m in cylindrical coordinates (resonant frequency from Figure 2d, the inverse ribbon width of the grating, 2π*n/w*, *n* = 6, and the twisting angle, $\varphi$) for the same Au grating twisted at different angles, $\varphi$. **b,d)** Isofrequency curves for the M1_m_ mode at different frequencies, *ω*. **c)** Analogous dispersion surface as in (a), but crossed by the planes of two fixed frequencies (*ω* = 908.5, and 911 cm^-1^). The red and black points mark the 6m peaks positions for Al gratings (Supplementary Figure 2a and Supplementary Figure 2b) in cylindrical coordinates (resonant frequencies, the inverse ribbon width of the grating, 2π*n/w*, *n* = 6, and the twisting angle, $\varphi$). The thicknesses of the flakes in (a,b) and (c,d) are 110 and 127.5 nm, respectively. |

In the main text probing the dispersion surface of the M0_a_ PhP mode (Figure 4) in the hyperbolic regime is performed for the 1a FPR in the area of the flake above air gaps. At the same time, the resonance 6m (the M1_m_ mode) in the area of the flake above the metal ribbons can also be achieved at the same frequency as the resonance 1a for all twisting angles, $\varphi$. Thus, the same resonant peaks in the reflection spectra (Figure 2d) can be interpreted both as the 1a FPR of the M0_a_ mode and the 6m FPR of the M1_m_ mode. Here, we perform probing the dispersion surface of the M1_m_ PhP mode (Supplementary Figure 4) in the hyperbolic regime on the example of the 6m FPR in the area of the flake above the metal ribbons. In Supplementary Figure 4a the experimentally-measured positions of the FPRs are represented as green, black, blue, and red points in cylindrical coordinates (the resonant frequency as a z coordinate, the inverse ribbon width, $2\pi n/w$, *n* = 6, as a radial coordinate and the twist angle, $\varphi$, as the polar angle). The purple cylinder with radius $2\pi n/w$, *n* = 6, represents the constant-momentum surface meaning that we perform the measurements for the same grating with fixed ribbon width, *w*, for all twist angles and frequencies, whereas the analytical dispersion surface of the M1_m_ mode is represented by a hyperboloid. The projections of the points from Supplementary Figure 4a on the (*k_x_, k_y_*) plane are positioned very close to the intersection points between the analytical isofrequency curves (IFCs) for the corresponding frequencies and the cylinder projection on the (*k_x_, k_y_*) plane (Supplementary Figure 4b).

An alternative probing of the PhP dispersion surface can be realized by constructing the cross-sections of the hyperboloid by constant-frequency planes (Supplementary Figure 4c), i.e. IFCs corresponding to the resonant frequencies in Supplementary Figure 2a,b. The projections of the experimentally-measured positions of the resonances, represented in cylindrical coordinates by red and black points in Supplementary Figure 4c, fit well the IFCs.

Our results indicate that the dispersion surface of the M1_m_ PhP mode in the hyperbolic frequency range can be experimentally reconstructed by measuring the resonance 6m in the region of the flake above the metal ribbons.

**Supplementary Note IV. Dispersion Surfaces of the M1_a_ and M1_m_ PhP Modes above both the Air Gap and the Metal Ribbon Regions in the α-MoO_3_ Elliptic Frequency Range**

| 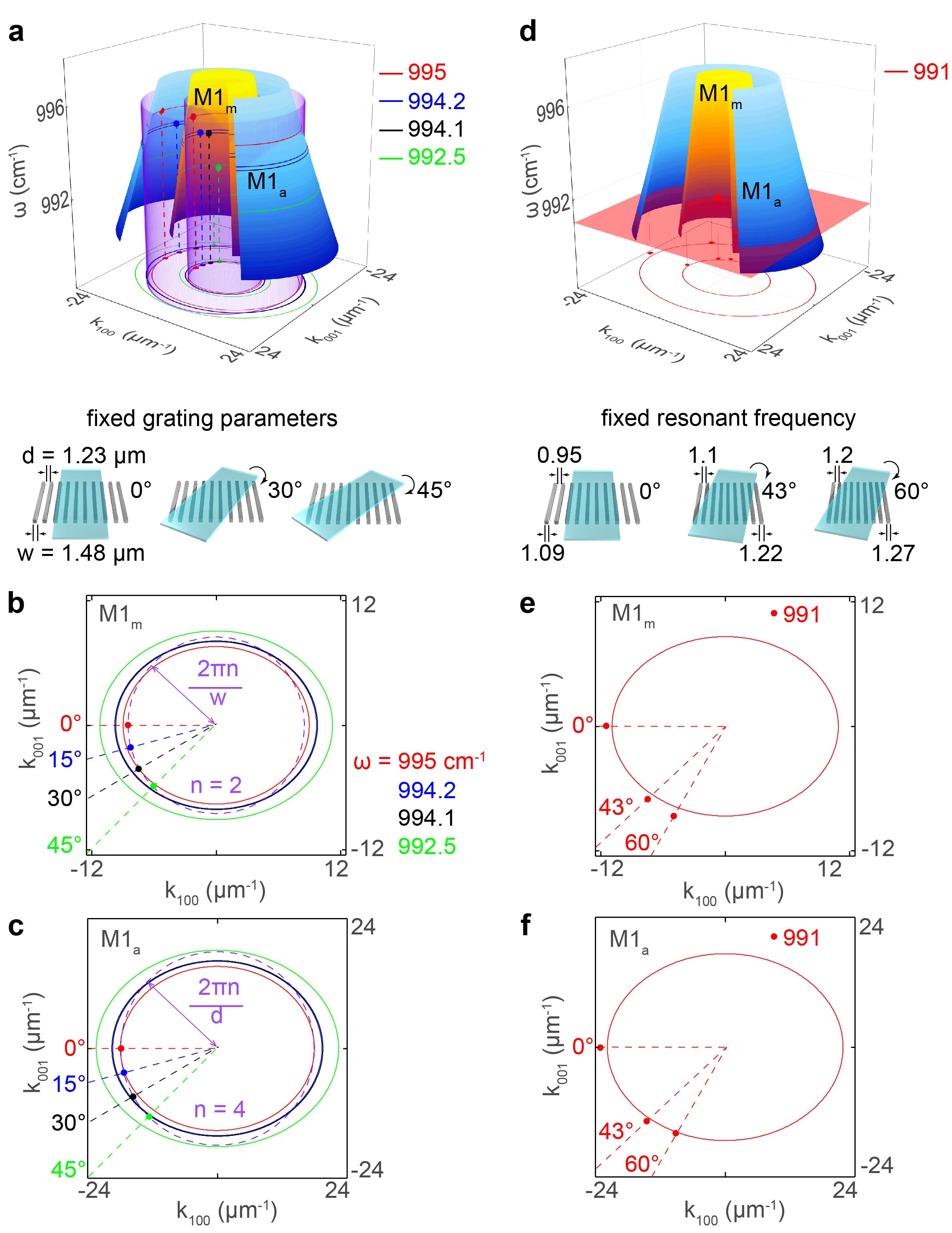 |
| --- |
| **Supplementary Figure 5.** **Probing the dispersion surfaces of the M1_a_ and M1_m_ PhP modes in the elliptic range. a)** Analytical dispersion surfaces of the M1_a_ and M1_m_ PhP modes crossed by two cylinders representing constant momenta. The color dots mark the positions of the measured FPR peaks, 2m (near the yellow surface of the M1_m_ PhP mode) and 4a (near the blue surface of the M1_a_ PhP mode) in cylindrical coordinates (resonant frequency from Supplementary Figure 3d; either the inverse ribbon width, 2π*n/w*, *n* = 2, or the inverse air gap width, 2π*n/d*, *n* = 4, for 2m and 4a resonances, respectively; and the twisting angle, $\varphi$) for the same Au grating twisted at different angles, $\varphi$. **b,e)** Isofrequency curves for the M1_m_ mode at different frequencies, . **c,f)** Isofrequency curves for the M1_a_ mode at different frequencies, . **d)** Analogous dispersion surface as in (a), but crossed by a plane of a fixed frequency, ω = 991cm^-1^. The points mark the 2m and 4a FPR peak positions for Al gratings (resonant frequencies from Supplementary Figure 2c, the inverse ribbon width of the grating, 2π*n/w*, *n* = 2, and the inverse air gap width of the grating, 2π*n/d*, *n* = 4, for 2m and 4a resonances, respectively, and the twisting angle, $\varphi$). The thicknesses of the flakes in (a,b,c) and (d,e,f) are 110 and 127.5 nm, respectively. |

In analogy to the results shown in Figure 4 of the main text for probing the dispersion surface of M0_a_ PhP mode in the hyperbolic range (performed for the 1a FPR emerging in the flake area above the air gaps), we represent in Supplementary Figure 5 the results of the probing of the dispersion surfaces of the M1_a_ and M1_m_ PhP modes in the elliptic range for the 4a and 2m FPRs in the flake area above the air gaps and the metal ribbons, respectively. The experimental data for Supplementary Figure 5a-c was taken from Supplementary Figure 3d, while the one for Supplementary Figure 5d-f was taken from Supplementary Figure 2c.

**Supplementary Note V. Experimental Quality Factors of the Nanoresonators**

**
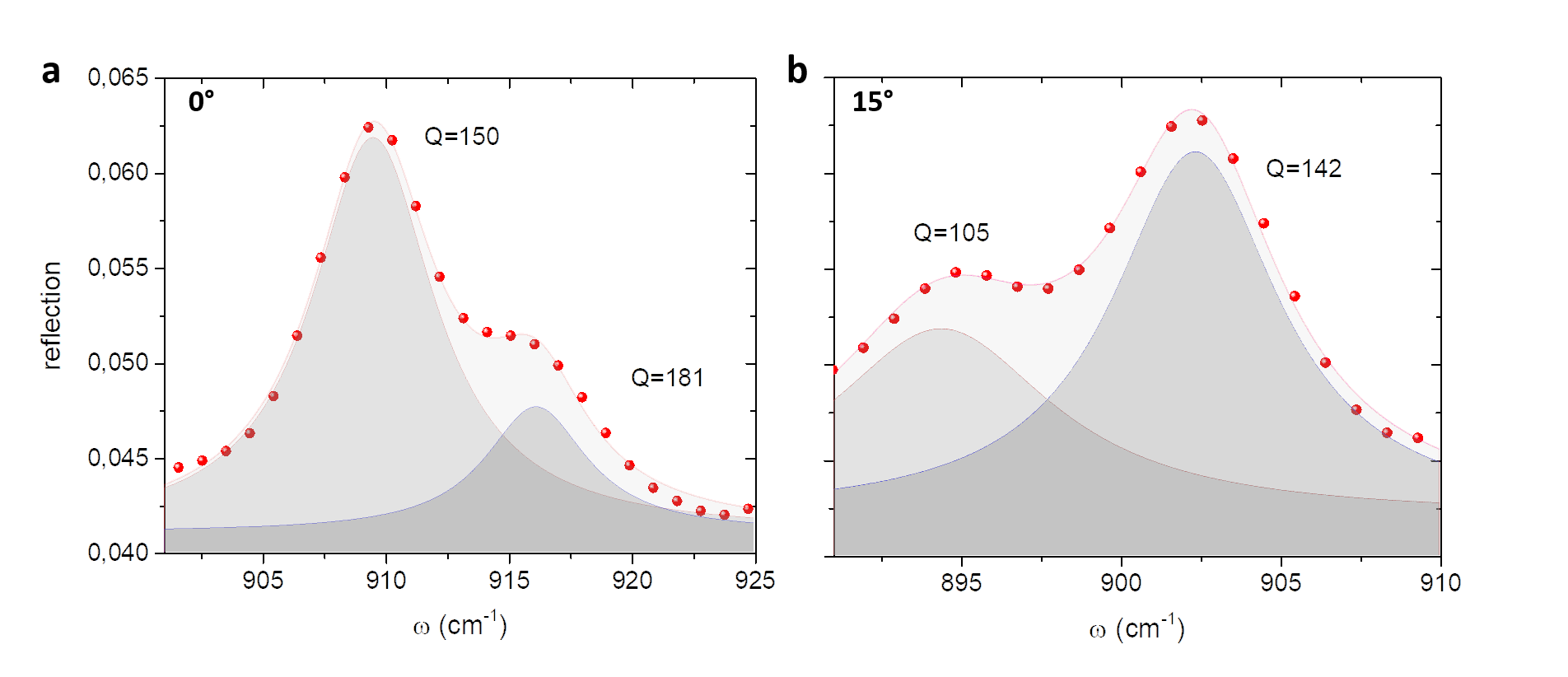
 Supplementary Figure 6.** **Fitting of the experimental reflection spectra in the hyperbolic range.** The α-MoO_3_ flake is placed on top of the metal grating with *w* = 1.48 μm and *d* = 1.23 μm. **a)** Rotation angle *φ* = 0º. **b)** Rotation angle *φ* = 15º.

To evaluate the quality factors (*Q*) of the nanoresonators, we choose resonant peaks corresponding to the 1a and 6m resonances in the α-MoO_3_ hyperbolic range and the 4a and 2m resonances in the elliptic range. In order to extract *Q*, we fit the reflection spectra by two Lorentzian curves. The red symbols represent the experimental data measured, while the gray areas represent the fitted Lorentzian line shape to the experimental spectra (Supplementary Figure 6). The quality factor can be defined as:

$Q= \frac{\omega_{res}}{\Delta\omega}$ (4)

where Q is the relation between the resonance frequency $\omega_{res}$ and the full width at half maximum Δω. We used the formula:

$y=y_{0}+ \frac{2A}{\pi}\frac{\Delta\omega}{4{(\omega-\omega_{res})}^{2}+{\Delta\omega}^{2}}$ (5)

obtaining the following results for the hyperbolic range:

| **Rotation angle  °** | $\boldsymbol{\omega}_{\mathbf{res}}$**  cm^-1^** | $\boldsymbol{\Delta\omega}$** cm^-1^** | **Quality factor (Q)** |
| --- | --- | --- | --- |
| **0** | **Peak 1** 909.50 **Peak 2** 916.14 | **Peak 1** 6.05 **Peak 2** 5.07 | **Peak 1** 150 **Peak 2** 181 |
| **15** | **Peak 1** 894.38 **Peak 2** 902.35 | **Peak 1** 8.56 **Peak 2** 6.37 | **Peak 1** 105 **Peak 2** 142 |
| **30** | 896.97 | 8.86 | 101 |
| **45** | 876.25 | 6.56 | 134 |

**Supplementary Table 1.** Quality factors (Q) for the hyperbolic range.

Analogously, we fitted (in this case by one Lorentzian curve) the resonances measured in the elliptic range obtaining the following values:

| **Rotation angle  °** | $\boldsymbol{\omega}_{\mathbf{res}}$**  cm^-1^** | $\boldsymbol{\Delta\omega}$** cm^-1^** | **Quality factor (Q)** |
| --- | --- | --- | --- |
| **0** | 995.16 | 7.07 | 141 |
| **15** | 994.76 | 12.66 | 79 |
| **30** | 994.56 | 14.56 | 68 |
| **45** | 992.76 | 4.99 | 199 |

**Supplementary Table 2.** Quality factors (Q) for the elliptic range.

**Supplementary References**

1. Nikitin, A. Y., Low, T. & Martín-Moreno, L. Anomalous reflection phase of graphene plasmons and its influence on resonators. *Physical Review B*, **90**, 041407 (2014).
2. Álvarez-Pérez, G., Voronin, K. V., Volkov, V. S., Alonso-González, P. & Nikitin, A. Y. Analytical approximations for the dispersion of electromagnetic modes in slabs of biaxial crystals. *Physical Review B*, **100**, 235408 (2019).
